# Perineal Self-Acupressure’s Mechanism of Action

**DOI:** 10.1007/s11606-015-3194-9

**Published:** 2015-02-10

**Authors:** Ryan Abbott, Ka-Kit Hui

**Affiliations:** 1 Department of Medicine, David Geffen School of Medicine at UCLA, Los Angeles, CA USA; 2 Southwestern Law School, Los Angeles, CA USA

To the Editor: We appreciate Dr. Olson raising these issues and bringing additional perspective to perineal self acupressure’s mechanism of action. The site at which study participants were directed to apply pressure is indeed an acupressure point, although it is Huiyin or CV 1 (the point between the genitals and the anus), not Changquian or GV 1 (the point between the coccyx and the anus).^1^ Huiyin is used in Traditional Chinese Medicine (TCM) not only to treat constipation, but also to treat a variety of conditions including impotence, hemorrhoids, rectal prolapse and dysmenorrhea.^1^

Given that JGIM is a physician-oriented publication, we elected to avoid using TCM nomenclature to prevent confusion, and we selected a treatment with a mechanism of action that has been described in the conventional medical literature. That is not to say that perineal self-acupressure does not also work as a result of its neurostimulatory or anti-inflammatory effects, or through activation of endogenous opioid mechanisms.^2^^,^ 3 Electrical stimulation of the perineal and sacral regions is even being investigated by urologists to improve bladder function.^4^ In addition, perineal self-acupressure was an appealing candidate for study because while it can be used as part of a TCM-based treatment, it is also appropriate for discrete integration into a conventional medical practice.

At the UCLA Center for East West Medicine, we employ a physician-directed, team-based approach to patient care that integrates conventional medical care with TCM. Patients with constipation are treated holistically, which includes education in self-acupressure as well as trigger point stimulation and changes to diet and lifestyle. Readers interested in learning more about acupressure and TCM, or accessing instructional content and clinical tools for perineal self-acupressure, may visit www.exploreim.ucla.edu and http://perinealpressure.yale.edu.
